# The Neural Mechanisms of Private Speech in Second Language Learners’ Oral Production: An fNIRS Study

**DOI:** 10.3390/brainsci15050451

**Published:** 2025-04-25

**Authors:** Rong Jiang, Zhe Xiao, Yihan Jiang, Xueqing Jiang

**Affiliations:** 1 Research Institute of International Chinese Language Education, Beijing Language and Culture University, Beijing 100083, China; 2 School of International Chinese Language Education, Beijing Normal University, Beijing 100875, China; 3 Cognitive Science and Allied Health School, Beijing Language and Culture University, Beijing 100083, China; 4 Institute of Life and Health Sciences, Beijing Language and Culture University, Beijing 100083, China; 5 Key Laboratory of Language and Cognitive Science, Ministry of Education, Beijing Language and Culture University, Beijing 100083, China; 6 Center for the Cognitive Science of Language, Beijing Language and Culture University, Beijing 100083, China

**Keywords:** private speech, second language production, functional near-infrared spectroscopy, sociocultural theory

## Abstract

**Background**: According to Vygotsky’s sociocultural theory, private speech functions both as a tool for thought regulation and as a transitional form between outer and inner speech. However, its role in adult second language (L2) learning—and the neural mechanisms supporting it—remains insufficiently understood. This study thus examined whether private speech facilitates L2 oral production and investigated its underlying neural mechanisms, including the extent to which private speech resembles inner speech in its regulatory function and the transitional nature of private speech. **Methods**: In Experiment 1, to identify natural users of private speech, 64 Chinese-speaking L2 English learners with varying proficiency levels were invited to complete a picture-description task. In Experiment 2, functional near-infrared spectroscopy (fNIRS) was used to examine the neural mechanisms of private speech in 32 private speech users identified in Experiment 1. **Results**: Experiment 1 showed that private speech facilitates L2 oral production. Experiment 2 revealed that private and inner speech elicited highly similar patterns of functional connectivity. Among high-proficiency learners, private speech exhibited enhanced connectivity between the language network and the thought-regulation network, indicating involvement of higher-order cognitive processes. In contrast, among low-proficiency learners, connectivity was primarily restricted to language-related regions, suggesting that private speech supports basic linguistic processing at early stages. Furthermore, both private and outer speech showed stronger connectivity in speech-related brain regions. **Conclusions**: This is the first study to examine the neural mechanisms of private speech in L2 learners by using fNIRS. The findings provide novel neural evidence that private speech serves as both a regulatory scaffold and a transitional form bridging outer and inner speech. Its cognitive function appears to evolve with increasing L2 proficiency.

## 1. Introduction

### 1.1. Understanding Private Speech: Perspectives from Piaget and Vygotsky

Individuals often engage in the phenomenon of “speaking to themselves” while performing various tasks. This behavior, termed private speech, is typically low in volume and self-directed rather than socially oriented—that is, it is vocalized but not intended for interpersonal communication. Numerous studies have documented its occurrence across diverse domains, including language development [1], problem solving [2,3,4], planning and arithmetic [5,6], and emotional regulation [7]. Its widespread manifestation across multiple developmental contexts raises a fundamental question: Does private speech serve as a psychological tool, or is it merely a sign of cognitive immaturity?

The theoretical debate concerning the function of private speech dates back to the early 20th century, with sharply contrasting views from Piaget and Vygotsky. Piaget was the first to observe this phenomenon in children, which he termed “egocentric speech” [8]. He argued that such speech lacked communicative intent and would gradually fade as children’s cognitive capacities matured, ultimately giving way to socialized speech—a defining form of outer speech. From Piaget’s perspective, private speech signaled cognitive immaturity and a developmental inability to take the perspective of others.

In contrast, Vygotsky’s sociocultural theory presents a fundamentally different view. According to Vygotsky, private speech functions as a tool for self-regulation, particularly when individuals face tasks that exceed their current level of competence [9]. In this view, private speech serves as a scaffold for thought regulation. Vygotsky further conceptualized private speech as a transitional stage between outer speech and inner speech, the latter a form of unvocalized language used for internal thought [9]. As cognitive development progresses, private speech becomes progressively internalized, transforming into inner speech. Thus, private speech marks a critical phase in the internalization of language as a psychological tool for thought regulation.

Vygotsky’s perspective has received substantial empirical support. Research on problem solving [3], executive function development [2], and individual differences [6] has shown that children often use private speech to regulate their thoughts and support task performance, particularly when faced with challenges that exceed their current cognitive capabilities. Notably, private speech does not vanish with cognitive maturation. Adults and adolescents—despite having more fully developed cognitive systems—also engage in private speech when performing cognitively demanding tasks involving memory, attention, and higher-order thinking. Like children, they rely on private speech as a tool for thought regulation [10,11,12,13,14].

### 1.2. The Role of Private Speech in Adults’ Second Language Learning

Recent studies have examined whether private speech in adults retains the same scaffolding function described by Vygotsky, particularly within the context of second language (L2) learning [10]. Research has shown that L2 learners use private speech to support a range of tasks, including oral production [15,16] and reading comprehension [17]. Moreover, several studies suggest that the frequency of private speech tends to decrease as L2 proficiency increases, regardless of whether the task involves oral language use [11,18] or cognitively demanding domains, such as L2-based mathematics and logic [19]. This reduction is thought to reflect a developmental shift: as learners become more proficient, they progressively internalize the L2 into inner speech, reducing their reliance on overt private speech [11,18,19].

However, this pattern is not universally observed. While higher-proficiency learners may produce fewer instances of private speech related to task planning, self-evaluation, and memory storage, some studies report an overall increase in private speech frequency with growing proficiency [20,21]. These conflicting findings suggest that the relationship between proficiency and private speech may be more complex than a simple linear decline.

Broadly speaking, the regulatory role of private speech in thought is widely recognized, and L2 proficiency is regarded as a potential factor modulating its use in learning. Nevertheless, empirical research in this area remains limited. Most studies to date have relied on questionnaires or the textual analyses of private speech [14,18], offering indirect insights into its cognitive and functional underpinnings. Critically, the neural mechanisms that support private speech remain largely unexplored. Investigating these mechanisms could advance our understanding of both the nature and function of private speech in L2 learners. For instance, from a neurocognitive perspective, does private speech facilitate thought regulation in a manner analogous to inner speech? Furthermore, does it serve as a transitional form—exhibiting neural characteristics of both outer and inner speech—as proposed by Vygotsky [9]?

### 1.3. Neural Mechanisms of Private Speech: Insights from Inner and Outer Speech

Investigating the neural mechanisms of private speech poses considerable challenges due to its spontaneous, fragmented nature. To date, no study has directly examined these mechanisms, leaving important gaps in our understanding of the cognitive and functional underpinnings of private speech, as discussed above. Nevertheless, research on inner speech and outer speech provides a valuable foundation for inference, given that private speech shares features with both. Like inner speech, private speech is closely linked to thought regulation—a function emphasized in sociocultural theory. Meanwhile, like outer speech, it engages audible articulation.

Neuroimaging studies have identified several key brain regions involved in inner speech, particularly those associated with language processing, including the left superior temporal gyrus (STG) [22], left inferior frontal gyrus (IFG) [23], and left middle temporal gyrus (MTG) [22]. Additionally, motor-related regions, such as the supplementary motor area and premotor cortex, have also been implicated [24]. Compared to inner speech, outer speech engages many of the same language-related areas but with varying activation strengths, including Broca’s area [25], motor areas [25,26], and the supramarginal gyrus (SMG) [26,27].

Given that private speech encompasses characteristics of both inner and outer speech, its neural correlates are likely to involve overlapping regions implicated in both. Moreover, if private speech in L2 learners supports self-regulation in a manner similar to inner speech, one would expect its functional connectivity pattern to more resemble that of inner speech. Specifically, this would involve strengthened connectivity between the language network and higher-order regions such as the dorsolateral prefrontal cortex (DLPFC).

### 1.4. The Current Study

Despite growing evidence for the facilitative role of private speech in L2 learning, substantial gaps remain in the literature. First, many existing studies have relied on subjective measures—such as questionnaires or textual analysis—rather than direct observation or the experimental manipulation of private speech’s regulatory function. Second, and more critically, the neural mechanisms underlying private speech in L2 learners remain entirely unexplored, limiting our understanding of how private speech supports thought regulation at the neural level.

Therefore, the present study investigated the behavioral and neural mechanisms of private speech in L2 learners through two experiments. Both experiments employed a picture-description task, a well-established paradigm for eliciting private speech in L2 contexts [28,29,30].

The aim of Experiment 1 was to identify L2 learners who naturally engage in private speech during oral production, as this behavior cannot be reliably predicted in advance. We also examined whether private speech facilitated L2 oral performance in these learners. Given our interest in the influence of L2 proficiency, we also assessed whether proficiency modulates the effect of private speech on task performance. We hypothesized that private speech would enhance L2 oral production and that the magnitude of these effects would vary with learners’ proficiency levels.

Participants identified as private speech users in Experiment 1 were invited to participate in Experiment 2 one week later. The primary aim of Experiment 2 was to investigate the neural mechanisms underlying private speech in L2 learners who had demonstrated its use. Specifically, we sought to examine whether private speech supports thought regulation, as proposed by Vygotsky, and whether it exhibits transitional properties between outer and inner speech. To address these questions, we directly compared the neural correlates of private speech, inner speech, and outer speech.

We hypothesized the following: (1) If private speech functions as a mechanism for thought regulation during L2 oral production, it should recruit brain regions associated with both the language network and the thought-regulation network. (2) Given their shared role in cognitive self-regulation, the neural pattern of private speech should closely resemble that of inner speech, consistent with Vygotsky’s theoretical framework. (3) If private speech constitutes a transitional form between outer and inner speech, its neural pattern should not only resemble that of inner speech but also partially overlap with that of outer speech. (4) Learners with different levels of L2 proficiency may exhibit distinct neural patterns during private speech, reflecting developmental differences in the internalization and functional use of language.

To test these hypotheses, we employed functional near-infrared spectroscopy (fNIRS), a non-invasive neuroimaging technique that offers distinct advantages over both electroencephalography (EEG) and functional magnetic resonance imaging (fMRI). EEG is highly susceptible to artifacts caused by eye movements and facial or neck muscle activity and has limited spatial resolution. While fMRI provides high spatial precision, it is extremely sensitive to head and mouth movements and requires participants to remain in a confined and restrictive environment—conditions that are not conducive to natural speech production. In contrast, fNIRS offers sufficient spatial resolution to localize cortical activity, is more tolerant of movement artifacts, and allows for data collection in more ecologically valid, naturalistic settings. These features make fNIRS particularly well suited for studying private speech during real-time oral language tasks.

As a preview, our preliminary findings revealed that private speech facilitated L2 oral production, while its inhibition hindered performance. Critically, the strong similarity in functional connectivity patterns between private speech and inner speech supported the hypothesis that private speech serves as a tool for thought regulation. At the same time, the partial overlap in connectivity between private speech and outer speech underscored its transitional role between the two forms. Moreover, the connectivity pattern of private speech varied with proficiency: in lower-proficiency learners, private speech primarily supported language processing, whereas, in higher-proficiency learners, it additionally facilitated non-linguistic thought regulation, reflecting a developmental shift in its cognitive function.

## 2. Experiment 1: The Impact of Private Speech on L2 Oral Production

### 2.1. Objectives of Experiment 1

The primary objectives of Experiment 1 were twofold: (1) to identify which L2 learners of English naturally engaged in private speech during preparation for oral production, and (2) to examine whether private speech facilitated subsequent L2 oral performance, as well as whether this effect was modulated by L2 proficiency.

To address these aims, we recruited Chinese-speaking L2 learners of English representing both high and low proficiency levels. Participants completed a picture-description task under two conditions: a free private speech condition, in which private speech was permitted, and an inhibited private speech condition, in which its use was not allowed.

This study employed a 2 × 2 × 2 mixed factorial design. English proficiency (high vs. low) and private speech user status (user vs. non-user) served as between-subject factors, while private speech inhibition (free vs. inhibited) was treated as a within-subject factor.

### 2.2. Materials and Methods

#### 2.2.1. Participants

Participants were recruited from Beijing Language and Culture University, with the goal of enrolling 16 individuals in each of four experimental groups: (1) high-proficiency private speech users, (2) high-proficiency non-users, (3) low-proficiency private speech users, and (4) low-proficiency non-users. High-proficiency L2 learners were third- and fourth-year English majors who had scored above 70 on the Test for English Majors—Band 4. Low-proficiency learners were third- and fourth-year students from non-English majors, with College English Test—Band 4 scores ranging between 425 and 500.

To expedite recruitment, we initially preselected candidates by asking whether they commonly “talked to themselves”. Participants who responded “yes” were provisionally categorized as private speech users, while those who responded “no” were tentatively assigned to the non-user group. During the formal experiment, participants were monitored via video recording, and the experimenter simultaneously verified the presence or absence of private speech. Only participants who produced private speech (i.e., meaningful and intelligible vocalized utterances directed to oneself [31]) during the task were formally classified as private speech users.

Recruitment proceeded iteratively. For example, in the high-proficiency groups, once the target of 16 confirmed private speech users was reached, recruitment for that subgroup ceased, and efforts shifted to the remaining non-user subgroup. Among those recruited thereafter, if a participant provisionally classified as a non-user unexpectedly produced private speech during the experiment, their participation was discontinued after completing the first experimental block, and they were excluded from the final sample. This process continued until the required number of verified non-users was also reached.

A total of 88 participants were initially recruited. Of these, 24 were excluded from the final analysis, including 3 individuals who failed to complete the tasks conscientiously. Ultimately, 16 participants were successfully enrolled in each of the four experimental conditions (all females, aged 20–23). This gender composition reflects the predominantly female student population at the university and was also intended to minimize potential confounding effects of gender on the experimental outcomes.

Language proficiency was validated using China’s Standards of English Language Ability [32]. Significant differences were observed between the high- and low-proficiency groups across all measured domains—listening, reading, speaking, and writing—(*Fs*(1, 60) ≥ 12.002, *ps* < 0.01). No significant differences were found between private speech users and non-users, nor were there interactions between private speech status and English proficiency (*Fs*(1, 60) < 1, *ps* > 0.05), indicating successful implementation of the proficiency manipulation.

All participants provided informed consent prior to participation at the Language Acquisition and Cognition Laboratory, Beijing Language and Culture University, and received compensation upon completion of the experiment. Research protocol and materials were approved by the Ethics Committee of the University.

#### 2.2.2. Materials

The experimental materials were selected from the classic parent–child comic series *Father and Son*, chosen for its visual simplicity and narrative clarity, making it well suited for use in picture-description tasks. Three sets of materials were prepared, each consisting of a sequential storyline. Material 1, comprising four images, was used as a practice task to familiarize participants with the procedure. Materials 2 and 3, each containing six images, were used in the formal experimental blocks.

#### 2.2.3. Task and Procedure

**Figure 1:**
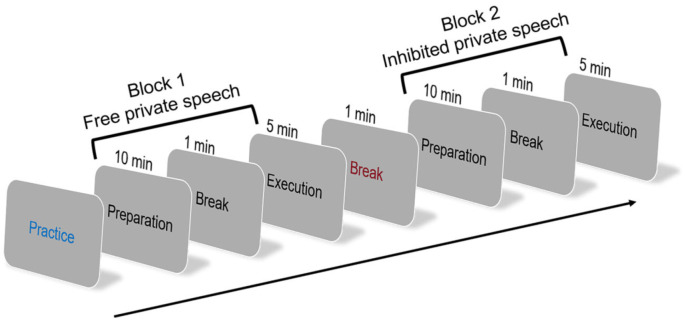
Experimental procedure of Experiment 1.

The procedure of Experiment 1 included a practice session followed by two formal blocks of a picture-description task (Figure 1). Each block consisted of two phases: a preparation phase and an execution phase. Participants completed the experiment individually in a soundproof room to ensure privacy, reduce external distractions, and promote the natural occurrence of private speech.

During the preparation phase, a sequence of images was presented on a computer screen. Participants were instructed to carefully plan their descriptions of the story depicted in the images, aiming to construct at least three English sentences per image with as much narrative detail as possible. Following the practice session and confirmation that participants fully understood the task instructions, they proceeded to the two formal experimental blocks. The preparation and execution phases were time-constrained to 10 and 5 min, respectively, with a one-minute rest interval.

Each participant completed two blocks of the picture-description task during the formal experiment: one under the free private speech condition and the other under the inhibited private speech condition. The entire experiment was audio- and video-recorded to monitor participants’ behavior and to assess the presence or absence of private speech during the task. In the free condition, participants were permitted to speak freely and were not required to wear any apparatus. In the inhibited condition, participants were instructed not to speak and were fitted with a mouth-breathing correction device designed to restrict jaw movement, thereby minimizing the possibility of articulation. This device does not cover the mouth, thereby allowing for clear observation of articulatory movements. Participants’ behavior was simultaneously monitored via video and audio recordings to ensure compliance with the instruction in this condition.

The free private speech condition was always administered first, allowing for the efficient identification and confirmation of private speech users. A one-minute break was provided between the two blocks. The order of picture sets was counterbalanced across participants. Specifically, half of the participants in each group completed Material 2 before Material 3, and the other half completed Material 3 before Material 2.

#### 2.2.4. Data Coding

**Preparation Phase (Private Speech Coding).** Data from the preparation phase were transcribed using an intelligent speech-to-text system and subsequently verified manually by a researcher, with a specific focus on identifying private speech during the free private speech condition. Private speech was defined according to the criteria established by Garbaj [33], which include vocalizations, whispers, and visible mouth movements. Examples include the following:

1. “em and in the……it is the tree, the tree, grow up, the tree……faster than the boy……shorter, than the record before”.
2. “等到了等到了春天还是夏天到了树叶再次茂盛的时候他们要站在这个树的下面像刚开始……似的……第一……的树然后发现钉子比他现在高了一截了” (When, when spring arrives, or summer arrives, the leaves grow lush again, they will stand beneath this tree, just like when they first……as the very first……tree, and then realize that the nail has risen a little higher than him).

For each participant, the following quantitative measures of private speech were calculated: total number of utterances (U), total number of words (W_PS, sum of English and Chinese words), average number of utterances per second (U/S), and average number of words per utterance (W/U). An utterance was defined as a complete sentence, sentence fragment, clause with a clear terminal marker, conversational turn, or any speech segment separated by a pause exceeding two seconds [34,35]. The number of English words was calculated automatically from the transcribed Microsoft Word documents. Chinese words were counted after segmentation using the Jieba Chinese word segmentation tool (https://github.com/fxsjy/jieba, accessed on 20 March 2023), and the results were manually reviewed by a researcher. If the researcher identified discrepancies or disagreed with the automated segmentation, the case was further discussed with a second researcher until consensus was reached. The number of seconds refers to the total cumulative duration of all time intervals in which private speech occurred.

**Execution Phase (Oral Production Coding).** Participants’ oral production was transcribed using an intelligent speech-to-text system and subsequently verified manually by a researcher. The quality of L2 oral production was assessed using complexity, accuracy, and fluency (CAF) indicators, which are widely used to evaluate L2 performance [36]. Fluency was measured through two indices: the total number of meaningful words (W_OP) and the number of analysis of speech (AS) units. An AS unit is defined as an independent clause with its associated subordinate clauses or an independent sub-clausal unit with related subordinate clauses [37]. Complexity was assessed via two metrics: the number of complex AS units (CAS), defined as AS units containing at least one dependent clause [37], and the average number of words per AS unit (W/AS), calculated as the ratio of total words to the number of AS units [38]. Accuracy was indexed by the total number of erroneous words (EWs) in each participant’s production, with a higher EW score indicating lower accuracy. All CAF indices were automatically computed using the L2 Syntactic Complexity Analyzer (https://aihaiyang.com/software/l2sca/, accessed on 9 April 2023), a validated English text analysis tool [39,40,41,42]. Disfluencies—such as false starts, repetitions, and self-corrections—were excluded prior to analysis.

### 2.3. Results

#### 2.3.1. Preparation Phase: Private Speech

During the preparation phase, participants classified as private speech users produced a comparable amount of private speech under the free private speech condition, irrespective of their L2 proficiency level (Table 1).

Two-sample *t*-tests revealed no significant differences between high- and low-proficiency learners on any private speech measures: total number of utterances, *t*(30) = −0.502, *p* = 0.619, Cohen’s *d* = 0.177; total number of words, *t*(30) = 0.401, *p* = 0.691, Cohen’s *d* = 0.142; average number of utterances per second, *t*(30) = −0.502, *p* = 0.619, Cohen’s *d* = 0.177; or average number of words per utterance, *t*(30) = 0.773, *p* = 0.446, Cohen’s *d* = 0.273.

#### 2.3.2. Execution Phase: Oral Production

A 2 (private speech user: yes/no) × 2 (inhibition: free/inhibited) × 2 (L2 proficiency: high/low) repeated-measures ANOVA was conducted for each of the CAF measures. Table 2 presents the means and standard deviations for each of the CAF measures.

**Fluency.** For W_OP, there was a significant interaction between inhibition and private speech user status, *F*(1, 60) = 5.320, *p* = 0.025, partial *η*^2^ = 0.081. Simple effect analysis revealed that, for private speech users, inhibition significantly reduced word production, *F*(1, 60) = 7.047, *p* = 0.010, partial *η*^2^ = 0.105. This medium-to-large effect indicates a notable influence of private speech, as users produced significantly more words in the free private speech condition (*M* = 305.656, *SD* = 101.616) than in the inhibited condition (*M* = 279.563, *SD* = 112.966). In contrast, no inhibition effect was found for non-users of private speech, *F*(1, 60) = 0.369, *p* = 0.546, partial *η*^2^ =0.006. No significant main effects or additional interactions were found, *Fs*(1, 60) < 3, *ps* > 0.1, partial *η*^2^s < 0.001.

For AS units, the interaction between inhibition and private speech user status was marginally significant, *F*(1, 60) = 2.923, *p* = 0.093, partial *η*^2^ = 0.046. Simple effect analysis showed that, for non-users of private speech, inhibition significantly increased the number of AS units produced, *F*(1, 60) = 4.165, *p* = 0.046, partial *η*^2^ = 0.065. Specifically, non-users produced more AS units under the inhibited condition (*M* = 24.688, *SD* = 8.422) than under the free condition (*M* = 22.656, *SD* = 6.617). For private speech users, no significant effect of inhibition was observed, *F*(1, 60) = 0.142, *p* = 0.708, partial *η*^2^ = 0.002. Although inhibition appeared to slightly increase syntactic complexity among non-users of private speech, the small-to-moderate effect sizes suggest that this influence was relatively modest. No significant main effects or additional interactions were found, *Fs*(1, 60) < 2, *ps* > 0.1, partial *η*^2^s < 0.03.

**Complexity.** For CAS, a significant interaction was observed between inhibition and private speech user status, *F*(1, 60) = 4.139, *p* = 0.046, partial *η*^2^ = 0.065. Simple effect analysis revealed a marginally significant effect of inhibition for private speech users, *F*(1, 60) = 2.855, *p* = 0.096, partial *η*^2^ = 0.045. Specifically, private speech users produced more complex AS units in the free condition (*M* = 7.375, SD = 4.094) than in the inhibited condition (*M* = 6.219, *SD* = 3.679). For non-users, no significant effect of inhibition was found, *F*(1, 60) = 1.410, *p* = 0.240, partial *η*^2^ = 0.023. Taken together with the small-to-moderate effect sizes, these results suggest that restricting private speech may modestly hinder the production of more syntactically complex utterances among private speech users. No significant main effects or other interactions were observed, *Fs*(1, 60) < 1, *ps* > 0.1, partial *η*^2^s < 0.02.

For W/AS, a significant main effect of L2 proficiency was found, *F*(1, 60) = 8.420, *p* = 0.005, partial *η*^2^ = 0.123. High-proficiency learners (*M* = 13.145, *SD* = 4.449) produced more words per AS unit than low-proficiency learners (*M* = 11.703, *SD* = 2.140). Additionally, the interaction between proficiency and private speech user status was significant, *F*(1, 60) = 4.528, *p* = 0.037, partial *η*^2^ = 0.070. Simple effect analysis indicated that, among private speech users, proficiency significantly influenced W/AS values, *F*(1, 60) = 12.648, *p* = 0.001, partial *η*^2^ = 0.174. High-proficiency users produced significantly more words per AS unit (*M* = 14.024, *SD* = 2.982) than low-proficiency users (*M* = 10.874, *SD* = 1.258). In contrast, no effect of proficiency was found for non-users, *F*(1, 60) = 0.299, *p* = 0.586, partial *η*^2^ = 0.005. These results, along with the large effect sizes, indicate that L2 proficiency has a strong influence on syntactic complexity in oral production, particularly among learners who engage in private speech. No other significant main effects or interactions were observed, *Fs*(1, 60) < 1, *ps* > 0.1, partial *η*^2^s < 0.02.

**Accuracy.** For the total number of wrong words (EWs), no significant main effects or interactions were found, *Fs*(1, 60) < 2, *ps* > 0.1, partial *η*^2^s < 0.03.

### 2.4. Discussion

This study investigated the effects of private speech on L2 learners’ oral production, yielding three key findings. First, there was no significant difference in private speech production between high- and low-proficiency learners. Second, private speech significantly enhanced oral fluency and complexity, particularly among learners who naturally engaged in it. Third, L2 proficiency exerted a strong influence on production complexity, with higher-proficiency learners producing structurally more complex speech. These findings offer valuable insights into the role of private speech in L2 learning.

Specifically, the absence of significant proficiency-based differences in private speech use during the preparation phase challenges earlier claims that private speech declines with increasing proficiency [29]. Rather, this result aligns with Vygotsky’s original view that cognitive tools are context-dependent. According to Vygotsky [9], when tasks are cognitively demanding, learners—regardless of proficiency—may engage in private speech to support thought regulation. In the current study, the picture-description task required learners to integrate multiple types of information, including visual cues, spatiotemporal sequencing, and linguistic encoding in the L2. The demands of the task posed substantial challenges, even for high-proficiency learners, prompting reliance on private speech as a regulatory mechanism. This suggests that the use of private speech is not determined solely by proficiency level but is also shaped by task complexity. Consequently, L2 educators should not assume that more proficient learners have fully internalized their language as a psychological tool that renders private speech unnecessary. Instead, learners at all levels should be encouraged to employ private speech strategically in response to cognitive demands, rather than be discouraged from doing so based on proficiency alone.

A key finding of Experiment 1 is that allowing private speech during the preparation phase improved learners’ subsequent oral fluency and complexity, as evidenced by increased word output and a higher number of words per AS unit. These results suggest that adult L2 learners continue to depend on private speech as an explicit scaffold during language production. Inhibiting or restricting private speech appears to hinder L2 performance—particularly in fluency—which is consistent with previous findings [10]. This outcome supports prior research within the sociocultural theory framework [29,30], which posits that private speech enables learners to organize and regulate their language output more effectively. Therefore, pedagogically, allowing learners to use private speech in classroom settings may enhance their oral performance, particularly in terms of fluency and syntactic complexity, while the benefit appears especially pronounced for fluency, as reflected by its comparatively larger effect size.

It is worth noting that the facilitative effects of private speech on fluency and complexity were found exclusively among learners who naturally used private speech. When these learners were permitted to speak freely, they exhibited greater word output and produced more structurally complex utterances, suggesting that private speech helped them coordinate linguistic planning and execution more effectively. In contrast, for non-private speech users, inhibiting private speech did not significantly affect fluency. These learners likely do not rely on private speech for thought regulation, and, thus, the inhibition had minimal impact. Interestingly, preventing private speech use in this group appeared to slightly improve fluency—albeit weak in effect size—possibly because the additional constraint helped focus their attention more directly on the task. This differentiation between private speech users and non-users suggests that the effect of private speech is closely tied to individual cognitive strategy preferences. Nevertheless, the facilitative effect of inhibiting private speech in non-private speech users was limited to oral fluency. In contrast, for private speech users, the free use of private speech enhanced both fluency and complexity, suggesting broader benefits of private speech as a psychological tool for L2 learners.

Furthermore, higher L2 proficiency was associated with greater complexity in learners’ oral production, particularly among private speech users. Among all examined variables, L2 proficiency yielded the largest effect sizes, highlighting its substantial influence on complexity in L2 oral production. This finding is consistent with previous research showing that increased language proficiency facilitates the use of more advanced grammatical structures and vocabulary in oral production [43,44]. Notably, the effect of proficiency was especially pronounced among learners who engaged in private speech. Higher proficiency may enable these learners to more effectively utilize private speech as a psychological tool for thought regulation. In contrast, learners who do not habitually use private speech may lack access to this regulatory mechanism, limiting their capacity to enhance performance through self-directed language use.

Moreover, private speech did not significantly affect the accuracy of oral production, suggesting that, while private speech facilitates fluency and complexity, its influence on accuracy may be less pronounced. This outcome may be partially attributable to the limited sample size in the present study. Additionally, prior research indicates that L2 learners often face challenges in simultaneously managing all three dimensions of CAF during production tasks [45]. When learners prioritize fluency and complexity, their ability to maintain high levels of accuracy may be constrained by their current proficiency and limited cognitive and linguistic resources. This challenge is especially salient under time pressure and in tasks that impose high cognitive load [46]. Nevertheless, the results indicate that private speech users did not show any performance benefit when their private speech was inhibited, relative to when they were allowed to speak freely. This finding suggests that there was no apparent trade-off among the CAF dimensions for private speech users: the use of private speech improved fluency and complexity without compromising accuracy.

Due to contextual constraints in participant recruitment and our intention to minimize potential gender-related confounds, all participants in this study were female. However, recent research has shown that gender does not significantly influence private speech production in adult L2 learners [17]. Therefore, the potential impact of single-gender sampling on the generalizability of our findings is likely limited. Nonetheless, we encourage future studies to examine gender-related differences more systematically to determine whether they may play a role in private speech.

In sum, Experiment 1 demonstrated the facilitative effect of private speech on L2 learners’ oral performance, offering empirical support for a core tenet of sociocultural theory: private speech functions as a psychological tool for thought regulation during complex tasks. However, behavioral data alone are insufficient to elucidate the mechanisms through which private speech exerts its regulatory function, particularly its relation to inner speech and the integration of language and higher-order cognitive processes. To address these limitations, Experiment 2 employs fNIRS to investigate the neural mechanisms underlying private speech in L2 learners.

## 3. Experiment 2: Investigating the Neural Mechanisms of Private Speech in L2 Oral Production

### 3.1. Objectives of Experiment 2

Building on the findings of Experiment 1, which identified private speech users and demonstrated the facilitative effect of private speech on L2 oral production, Experiment 2 aimed to investigate the neural mechanisms underlying private speech in L2 learners using fNIRS. Participants were those previously identified as private speech users, and the same picture-description task was employed to ensure continuity across behavioral and neural measurements.

The primary objective was to examine the neurocognitive underpinnings of private speech. Given its theoretical proximity to both inner speech and outer speech, comparisons across these speech types were essential to delineate the unique neural characteristics of private speech. Furthermore, in light of prior findings highlighting the role of L2 proficiency in modulating private speech use, we also investigated whether proficiency levels influence the neural representation of private speech.

To address these aims, we employed a 2 (L2 proficiency: high/low) × 3 (speech type: private speech/inner speech/outer speech) mixed factorial design, with speech type as the within-subject factor. Speech conditions were operationally defined based on the experimental phases. Private speech segments were those during the preparation phase in which participants overtly produced self-directed speech (same as in Experiment 1), inner speech segments referred to preparation segments in which no overt speech was observed, and outer speech was represented by the execution phase, during which participants verbally described the story aloud.

### 3.2. Materials and Methods

#### 3.2.1. Participants

Participants in Experiment 2 were drawn from those identified as private speech users in Experiment 1. Consistent with the sampling characteristics of the previous experiment, all participants were female, aged 20–23. A total of 32 participants completed this study, with an equal number of high- and low-proficiency learners (*n* = 16 per group). All participants were right-handed and reported no history of neurological or psychiatric disorders. The experiment was conducted one week after the completion of Experiment 1. All participants provided informed consent prior to participation at the Language Acquisition and Cognition Laboratory of Beijing Language and Culture University and received compensation upon completion of this study. Research protocol and materials were approved by the Ethics Committee of the University.

#### 3.2.2. Materials

Two new sets of *Father and Son* comic strips were selected for use in Experiment 2. Each set consisted of six sequential images forming a coherent narrative. These materials were distinct from those used in Experiment 1.

#### 3.2.3. Task and Procedure

Participants completed a picture-description task similar to that used in Experiment 1. The experiment was conducted in a sound-attenuated room, where each participant was seated alone throughout the session. At the beginning of the experiment, participants received standardized instructions detailing the task procedures and requirements.

**Figure 2:**
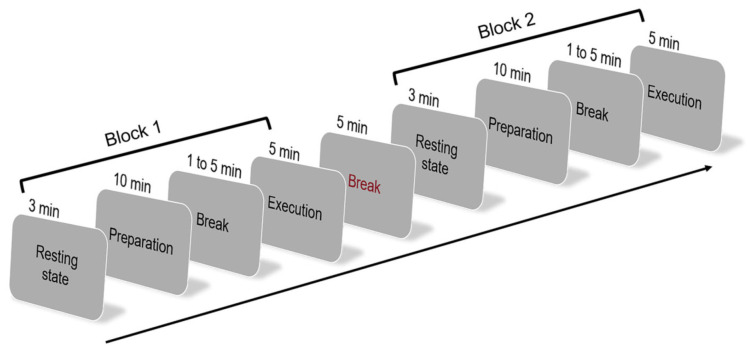
Experimental procedure of Experiment 2.

The experiment consisted of two picture-description blocks, each comprising a resting-state phase followed by a task phase (Figure 2). The resting-state phase lasted 3 min, during which participants were instructed to remain still, awake, and relaxed without engaging in any specific cognitive activity. This phase served as a baseline for assessing task-related neural activity. Each task phase included a preparation phase and an execution phase, following similar instructions and structure as in Experiment 1. A rest interval of 1–5 min was provided between the preparation and execution phases within each block, and a 5 min rest was given between the two picture-description blocks. Picture set order was counterbalanced using the same procedure as in Experiment 1. All sessions were audio- and video-recorded.

#### 3.2.4. Data Acquisition

fNIRS data were collected using ETG-7100 optical imaging system (Hitachi, Tokyo, Japan), which continuously measures concentrations of oxygenated hemoglobin (HbO), deoxygenated hemoglobin (HbR), and total hemoglobin (tHb) using near-infrared light at wavelengths of 695 nm and 830 nm. Data were sampled at a frequency of 10 Hz. Hemoglobin concentration changes were calculated using the modified Beer–Lambert law. Consistent with prior research [47], we focused primarily on the HbO signal, as it is more sensitive to task-related neural responses.

**Figure 3:**
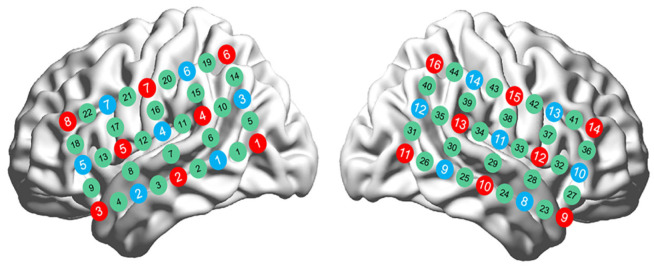
fNIRS optode probe set and channel placement, with red circles indicating emitters, blue circles indicating detectors, and green circles indicating channels.

For each participant, two 3 × 5 probe arrays were placed bilaterally on the scalp. Each array included 8 emitters and 7 detectors, yielding 22 channels per hemisphere (44 channels in total). The sensor placement and channel configuration are illustrated in Figure 3.

**Figure 4:**
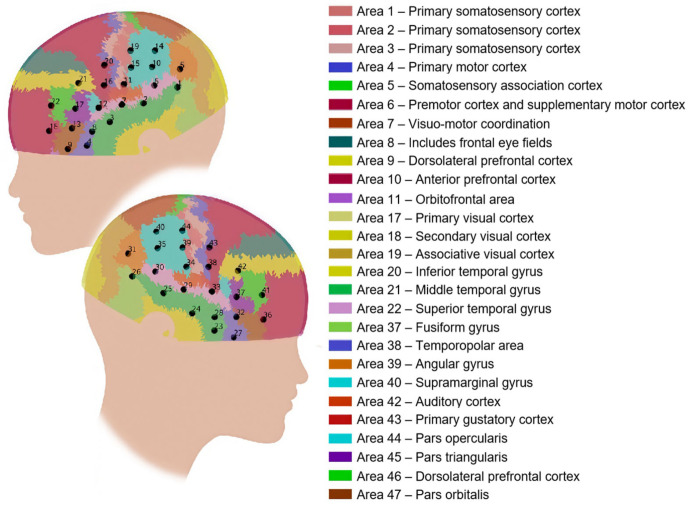
Corresponding brain regions and Brodmann area labels for all channels.

To obtain anatomical reference information, a 3D magnetic digitizer was used to record the scalp coordinates of each optode. These data were aligned to a standardized transcranial brain atlas [48], enabling estimation of the corresponding MNI coordinates and Brodmann areas for each channel at the group level (Figure 4). The recorded channels were found to encompass key brain regions associated with language processing (e.g., Broca’s and Wernicke’s areas), higher-order cognitive functions (e.g., the temporoparietal junction, TPJ), and motor regions implicated in speech production. Channels 4, 9, 23, and 27 were excluded from subsequent analyses, as localization results indicated that they fell outside the cranial boundary. This left 40 valid channels for further data analysis.

#### 3.2.5. Data Preprocessing

Signal quality assessment was performed using the sliding time-window method [49]. For each participant and each channel, HbO concentrations were averaged within a 5 s time window, and the standard deviation was computed. Time points with HbO values exceeding three standard deviations from the mean within that window were marked as artifacts. The proportion of artifact-labeled time points was then calculated for each channel. Channels with more than 5% artifact contamination were classified as low-quality and excluded from further analysis. Additionally, channels displaying poor signal quality—such as excessive noise, large signal spikes (particularly when HbO and HbR changed in the same direction), or signal dropout—were visually inspected and excluded.

Next, raw fNIRS data were preprocessed using MATLAB R2014a with custom scripts. The Temporal Derivative Distribution Repair algorithm was applied to remove head motion artifacts [50]. A high-pass filter with a cutoff frequency of 0.0078 Hz, based on discrete cosine trans-forms, and a low-pass filter based on the hemodynamic response function were used for filtering [51]. These preprocessing procedures were conducted individually on each participant’s data.

The preprocessed fNIRS time series were then segmented based on behavioral annotations. During the preparation phase, time segments were manually labeled as either private speech or inner speech, based on participants’ behavior observed in the audio and video recordings. The onset and offset time points of each segment were annotated. Segments in which participants produced self-directed speech were labeled as private speech, following the same criteria used in Experiment 1. Segments in which no overt articulation occurred—indicating silent, internally directed verbal thought—were labeled as inner speech. The execution phase was categorized as the outer speech condition, during which participants orally described the image sequences.

#### 3.2.6. Data Analysis

We analyzed both task-related brain activation and functional connectivity to examine the effects of speech type (private, inner, outer) and L2 proficiency (high, low). At the individual level, a general linear model (GLM) was applied to estimate activation values (i.e., beta coefficients) for each channel under each speech condition. Additionally, pairwise functional connectivity between channels was calculated using Pearson’s correlation coefficients. At the group level, statistical analyses focused on contrasts of interest (e.g., private speech vs. inner speech) to determine whether private speech elicited distinct neural responses. Group-level comparisons were conducted using *t*-tests. Detailed procedures for activation and connectivity analyses are described below.

**Activation analysis.** We first computed individual-level task-related activation values. The experiment included four conditions: resting state, private speech (during the preparation phase), inner speech (also during the preparation phase), and outer speech (i.e., the execution phase). For each condition, activation values were estimated from the HbO signal using a GLM applied for each participant and each channel.

At the group level, we performed channel-by-channel analyses to examine contrasts of interest. For each proficiency group, the following contrasts were examined: (1) Private speech > resting state (to assess activation related to private speech). (2) Inner speech > resting state (to assess activation related to inner speech). (3) Outer speech > resting state (to assess activation related to outer speech). (4) Private speech > inner speech (to assess differential activation between private and inner speech). In addition, we compared high- vs. low-proficiency learners to examine proficiency-related effects. One-sample *t*-tests were performed on individual task-related activation estimates to derive group-level activation for each channel and each contrast. Activation was considered significant if it surpassed a channel-level threshold of *p* < 0.05, with correction for multiple comparisons using the false discovery rate (FDR) method.

**Functional connectivity analysis.** To assess functional connectivity, we first computed individual-level 40 × 40 channel-wise functional connectivity matrices for each of the four experimental conditions using Pearson’s correlation. Correlation coefficients were calculated between the HbO time series of every channel pair, providing a measure of connectivity strength. To facilitate interpretation, the order of channels in each connectivity matrix was reorganized by grouping channels according to their corresponding brain regions of interest, including the frontal lobe, motor area, parietal lobe, and temporal lobe. The channel (CH) groupings were as follows: (1) Left frontal lobe (Channels 1–6): CH 12, CH 17, CH 21, CH 13, CH 18, CH 22. (2) Left motor area (7–9): CH 11, CH 16, CH 20. (3) Left parietal lobe (10–14): CH 5, CH 10, CH 15, CH 14, CH 19. (4) Left temporal lobe (15–20): CH 2, CH 6, CH 7, CH 1, CH 3, CH 8. (5) Right frontal lobe (21–25): CH 37, CH 42, CH 36, CH 41, CH 32. (6) Right motor area (26–29): CH 38, CH 43. (7) Right parietal lobe (30–33): CH 34, CH 39, CH 31, CH 35, CH 40, CH 44. (8) Right temporal lobe (34–40): CH 29, CH 30, CH 33, CH 24, CH 25, CH 26, CH 28. Each participant’s correlation matrices were Fisher-*Z*-transformed to improve normality and then averaged across the two picture-description blocks, resulting in one functional connectivity matrix per condition per participant.

At the group level, differences in functional connectivity between conditions were analyzed by generating three contrast matrices: (1) Private speech > outer speech. (2) Inner speech > outer speech. (3) Private speech > inner speech. For each contrast, paired-sample *t*-tests were performed for each connection across conditions to obtain *t*-values and *p*-values. The negative base-10 logarithm of the *p*-values, –log_10_(*p*), was calculated to create a transformed matrix indicating the strength of functional connectivity differences, where higher values denote more robust effects. No connections survived correction for multiple comparisons using false discovery rate (FDR)—likely due to the large number of comparisons. Therefore, we report uncorrected significant connections at *p* < 0.005.

### 3.3. Results

#### 3.3.1. Activation Analysis

**Figure 5:**
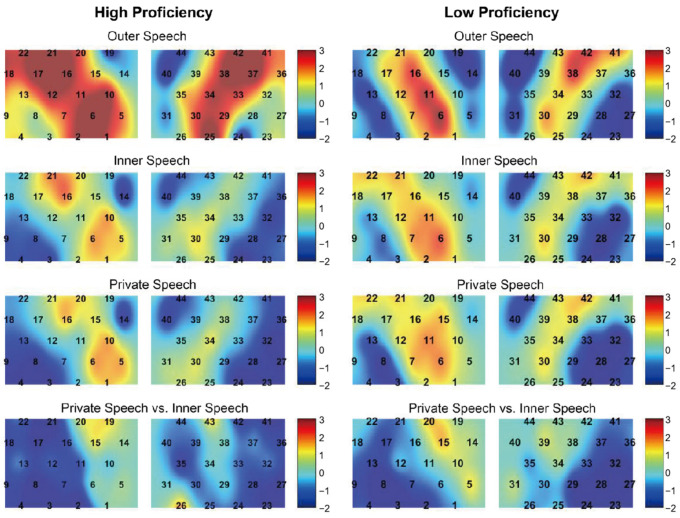
The *t*-value distribution maps for the four contrasts in high- and low-proficiency participants.

Figure 5 presents the *t*-value distribution maps for task-related activation across four contrasts in high- and low-proficiency participants. Overall, the spatial activation patterns associated with private speech, inner speech, and outer speech were broadly similar. Compared to inner speech, private speech elicited slightly stronger activation in motor areas, whereas other brain areas showed reduced activation. In contrast, outer speech (corresponding to the execution phase) produced stronger and more widespread activation than both private and inner speech, with more pronounced bilateral activation patterns.

Among high-proficiency learners, no significant activation was observed during either private speech or inner speech (corrected *ps* > 0.05), and no significant activation differences were found between the two conditions (corrected *ps* > 0.05). However, during outer speech, significant activation was observed in several regions, including the bilateral MTG (CH 1, *t* = 2.55, corrected *p* = 0.046; CH 25, *t* = 3.83, corrected *p* = 0.013), Broca’s area (CH 12, *t* = 3.02, corrected *p* = 0.033; CH 17, *t* = 3.41, corrected *p* = 0.024; CH 21, *t* = 3.39, corrected *p* = 0.016; CH 37, *t* = 3.52, corrected *p* = 0.013; CH 42, *t* = 3.60, corrected *p* = 0.013), left Wernicke’s area (CH 2, *t* = 3.18, corrected *p* = 0.030; CH 6, *t* = 4.45, corrected *p* = 0.013), the SMG (CH 10, *t* = 3.05, corrected *p* = 0.030), the junction of the SMG and motor area (CH 34, *t* = 2.83, corrected *p* = 0.038), bilateral motor areas (CH 16, *t* = 3.54, corrected *p* = 0.013; CH 38, *t* = 3.22, corrected *p* = 0.032), and the left orbitofrontal cortex (OFC; CH 18, *t* = 2.80, corrected *p* = 0.040).

Among low-proficiency learners, no significant activation was observed during private speech or inner speech, nor were there significant activation differences between the two conditions. During outer speech, a significant deactivation was found in the SMG (CH 44: *t* = −3.32, *p* = 0.032).

Finally, proficiency effects within each speech type were examined, with no significant differences found between high- and low-proficiency learners (corrected *ps* > 0.05).

#### 3.3.2. Functional Connectivity Analysis

**Figure 6:**
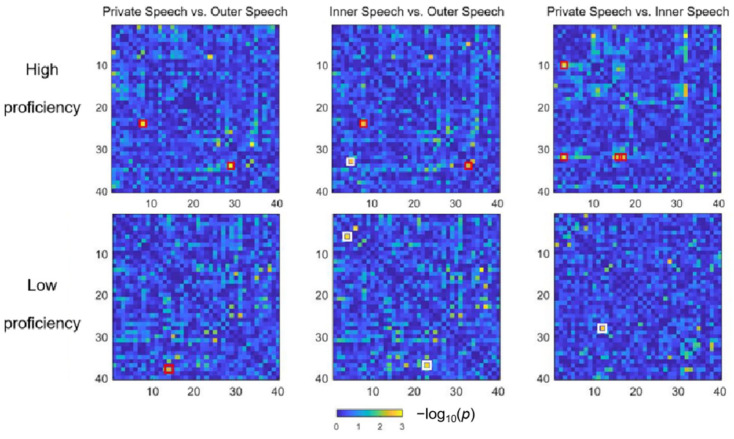
The *p*-value matrices for channel-wise functional connectivity across all three contrasts. Red boxes indicate significantly stronger functional connectivity, while white boxes indicate significantly weaker functional connectivity. The numbers on the horizontal and vertical axes represent rearranged channel numbers, as defined in the Data Analysis Section, rather than the original channel numbers.

Figure 6 presents the significant functional connectivity results for the three contrasts. Among high-proficiency learners, both private speech and inner speech exhibited significantly stronger functional connectivity compared to outer speech. Specifically, increased connectivity was observed between the left motor area and right DLPFC (CH 16–CH 41; private speech > outer speech: *t* = 3.48, *p* = 0.004; inner speech > outer speech: *t* = 3.38, *p* = 0.005) and between the right SMG–motor area junction and right STG (CH 39–CH 29; private speech > outer speech: *t* = 3.87, *p* = 0.002; inner speech > outer speech: *t* = 3.45, *p* = 0.004).

Additionally, compared to inner speech, private speech among high-proficiency learners exhibited significantly stronger connectivity between the left Broca’s area and angular gyrus (AG; CH 21–CH 5: *t* = 4.30, *p* = 0.0008), between the left Broca’s area and right SMG (CH 21–CH 40: *t* = 5.21, *p* = 0.0002), and between the left STG and right SMG (CH 6–CH 40: *t* = 3.75, *p* = 0.002; CH 7–CH 40: *t* = 3.48, *p* = 0.004). In contrast, outer speech exhibited significantly stronger connectivity between the left OFC and right SMG (CH 18–CH 44: *t* = 4.05, *p* = 0.001).

Among low-proficiency learners, a distinct connectivity pattern was observed. Com-pared to outer speech, private speech was associated with significantly stronger connectivity between the left SMG–motor area junction and right MTG (private speech > outer speech: CH 19–CH 25, *t* = 3.75, *p* = 0.002). Moreover, when compared to inner speech, private speech in low-proficiency learners exhibited significantly weaker connectivity between the left and right SMG–motor area junctions (CH 15–CH 34: *t* = 3.47, *p* = 0.004), while outer speech exhibited significantly stronger connectivity between the left pars orbitalis (orbital part of the IFG) and the left DLPFC–frontal pole junction (CH 13–CH 22: *t* = 3.72, *p* = 0.0025) and between the right frontal pole and right MTG (CH 36–CH 24: *t* = 4.13, *p* = 0.002).

### 3.4. Discussion

This study is the first to employ fNIRS to investigate the neural mechanisms underlying private speech in L2 learners. The functional connectivity analyses revealed three key findings. First, the connectivity pattern of private speech more closely resembled that of inner speech than outer speech, supporting its role as a cognitive scaffolding mechanism akin to inner speech. Second, despite this resemblance, private and inner speech exhibited distinct functional connectivity patterns, and private speech also shared connectivity features with outer speech, highlighting its transitional nature between outer and inner speech. Third, functional connectivity patterns differed between high- and low-proficiency learners, suggesting that the neural correlates supporting private speech are modulated by L2 proficiency and develop in tandem with linguistic expertise.

#### 3.4.1. Activation of Different Speech Types

Regarding neural activation, no significant group-level activation was observed during either private speech or inner speech, nor were there significant differences between these two conditions. In contrast, outer speech elicited widespread cortical activation. Consistent with previous findings, high-proficiency learners showed significant activation in multiple language-related regions, including bilateral activation in Broca’s area (CH 12, CH 17, CH 37, CH 42, CH 21) and left Wernicke’s area (CH 2, CH 6), both of which are critical for speech production and comprehension [52]. Additional activation was observed in the OFC (CH 18), associated with speech sound processing [53], motor areas (CH 16, CH 38), involved in speech execution, and the posterior MTG (CH 1, CH 25), linked to semantic memory and processing [54,55,56]. Furthermore, activation was identified in the left SMG (CH 10), associated with phonological working memory [57,58], and the right SMG, which is involved in lexical-level phonological representation and processing [59,60,61] and speech motor output [62]. These findings suggest that L2 oral production in high-proficiency learners engages a broad neural network that supports phonological and semantic processing, syntactic construction, and motor planning for speech. By contrast, low-proficiency learners exhibited significant deactivation in the right SMG, potentially reflecting difficulties in L2 lexical-level phonological representation and processing.

In the present study, private speech did not elicit significant neural activation, a result likely attributable to its transient and fragmented nature. As illustrated by Example 1 (e.g., “the tree, the tree”), private speech often consists of isolated lexical items or short, disjointed phrases, frequently lacking full syntactic structure. This discontinuity makes it difficult to detect consistent hemodynamic signals at the group level. In addition, individual variability in cognitive strategies may further obscure activation patterns. For instance, some learners may focus on lexical rehearsal, while others prioritize syntactic planning, leading to highly heterogeneous neural responses. Finally, as an exploratory study, the relatively modest sample size may have limited statistical power to detect stable activation associated with private speech. Future research should consider increasing the sample size to better elucidate the neural activation patterns underlying private speech.

#### 3.4.2. Private as a Thought-Regulation Tool Similar to Inner Speech

Importantly, private speech exhibited greater similarity functional connectivity patterns with inner speech than outer speech, suggesting that these two forms of speech may operate comparably within neural networks, particularly in relation to thought regulation. Especially, among high-proficiency learners, both private and inner speech were associated with increased connectivity between the left motor area and right DLPFC and between the right SMG–motor area junction and right STG. This pattern is consistent with previous findings showing that inner speech more strongly engages left motor areas and cognitive control regions than outer speech [63]. The left motor area is fundamental to speech production, controlling articulatory movements [64], while the DLPFC is involved in working memory, cognitive control [65], the inhibition of non-target language representations [66], auditory feedback monitoring [67,68], and higher-level discourse and prosodic integration [69]. These findings suggest that both mature private and inner speech play important roles in non-linguistic thought regulation by reinforcing the connection between language-related and thought-related networks. In addition, the right STG is involved in speech processing [70,71] and the right SMG in phonological processing [59,60,61]. Thus, both forms of speech not only promote integration between language and thought-regulation networks but also enhance intra-network coordination within the language system by coordinating speech perception and motor execution [72].

In contrast, low-proficiency learners displayed distinct functional connectivity patterns, indicating that L2 proficiency modulates the neural mechanisms supporting private speech. For these learners, private speech increased connectivity between the left SMG–motor area junction and right MTG. The left SMG is involved in lexical-level phonological processing [61], while the right MTG is associated with semantic memory [54]. Given that phonological representations in low-proficiency learners are still developing, private speech may function as a lexical support mechanism, aiding word retrieval by engaging phonological and semantic systems. This increased reliance on phonology likely strengthens connectivity between the SMG and MTG, without engaging prefrontal regions associated with discourse-level control or cognitive control. Thus, for low-proficiency learners, private speech primarily enhances within-network connectivity in the language processing system.

The functional connectivity findings discussed above raise several important theoretical considerations. First, our results indicate that mature private speech exhibits a functional profile closely aligned with that of inner speech. From the perspective of sociocultural theory, this provides neurobiological evidence supporting the role of private speech as a psychological tool for thought regulation. The observed similarity in functional connectivity patterns between private and inner speech, especially when compared with outer speech, aligns directly with Vygotsky’s conceptualization of private speech as a critical mechanism for self-regulation and cognitive scaffolding. These findings move beyond behavioral inference, offering direct neural support for the proposition that private speech operates in a manner comparable to inner speech in mediating internal thought processes.

Second, the differential connectivity patterns observed between high- and low-proficiency learners point to a developmental trajectory in the neural function of private speech. As L2 proficiency increases, private speech appears to facilitate increasingly complex integration between language systems and executive control networks, thereby enhancing cognitive regulation during speech planning and production. This pattern is consistent with prior behavioral research grounded in sociocultural theory, which suggests that L2 learners’ self-regulatory capacities develop alongside their growing linguistic competence [73]. Vygotsky characterized private speech as undergoing developmental transformation, and our findings extend this theoretical framework by elucidating the dynamic, proficiency-sensitive nature of private speech at the neural level. Specifically, the shift in connectivity within the language network to cross-network integration reflects a functional evolution in how private speech supports regulation. Rather than simply fading with increased proficiency, private speech appears to shift in function—from serving primarily as a linguistic scaffold to also facilitating higher-order, non-linguistic functions, much like inner speech. This perspective offers a more nuanced account of internalization, suggesting that private speech evolves in function as learners advance, thereby refining our understanding of its role in L2 development from a sociocultural perspective.

#### 3.4.3. Private Speech as a Transitional Form Bridging Outer and Inner Speech

Notably, while the functional connectivity of private speech in high-proficiency learners supports its role as a cognitive scaffold akin to inner speech, it also exhibits distinct features and shares neural characteristics with outer speech. Specifically, in high-proficiency learners, private speech showed stronger connectivity between the left Broca’s area and both the left AG and left SMG, as well as between the left STG and right SMG. The left AG is implicated in higher cognitive functions, including attention, motor planning [74], creative thinking [75], and access to prior knowledge [76], while the left STG plays a critical role speech processing, lexical–semantic processing, and auditory analysis [77,78]. The right SMG has been shown to undergo structural plasticity following L2 learning, underscoring its involvement in L2 processing [79]. Compared to inner speech, private speech thus appears to facilitate greater coordination between core language-processing regions (Broca’s area, SMG, STG) and higher-order cognitive hubs (e.g., AG), suggesting that private speech may serve additional regulatory functions beyond those supported by inner speech alone.

Moreover, private speech exhibited functional connectivity patterns similar to outer speech, particularly in phonological processing. Specifically, compared with inner speech, in high-proficiency learners, private speech showed stronger connectivity between the left STG and right SMG, while outer speech showed stronger connectivity between the left OFC and right SMG. This overlap suggests that private speech retains phonological processing characteristics typical of outer speech, thereby distinguishing it from the fully internalized nature of inner speech.

In contrast, low-proficiency learners did not exhibit this pattern. For them, private speech did not elicit stronger connectivity than inner speech; in fact, it showed weaker connectivity between the left and right SMG–motor area junction, implying that private speech may not yet confer additional scaffolding benefits beyond what is offered by inner speech at earlier stages of L2 development. Additionally, compared to inner speech, outer speech in low-proficiency learners was associated with enhanced connectivity between the right frontal pole and right MTG and between the left pars orbitalis and the left DLPFC–frontal pole junction. The involvement of these regions, which support phonological and motor aspects of speech production [80,81,82], suggests that low-proficiency learners rely more heavily on overt articulatory and auditory–motor processes during speech.

Taken together, the shared connectivity between private and inner speech, along with its neural resemblance to outer speech, supports the conceptualization of private speech as a transitional form between outer and inner speech. Our findings present a nuanced neural picture. On the one hand, private speech is not yet fully internalized inner speech, as it provides additional self-regulatory functions beyond those afforded by inner speech in high-proficiency learners while remaining less functionally mature in lower-proficiency learners. On the other hand, private speech retains certain characteristics of outer speech, as it continues to engage brain regions associated with overt speech processing. This provides neurobiological grounding for the role of private speech in the internalization of language as a psychological tool, as posited by sociocultural theory.

#### 3.4.4. Summary and Pedagogical Implications

In conclusion, this study is the first to employ fNIRS to investigate the neural mechanisms underlying private speech in L2 learners, offering novel insights into its role as a psychological tool for thought regulation in the context of L2 oral production. The results provide neurobiological support for Vygotsky’s hypothesis that private speech functions both as a regulatory mechanism akin to inner speech and as a transitional stage between outer and inner speech. Furthermore, the data suggest that, with increasing L2 proficiency, private speech evolves from supporting basic linguistic processing to facilitating higher-order, non-linguistic thought regulation, thereby enhancing functional connectivity between language and thought-regulation networks.

These findings yield several important pedagogical implications for L2 instruction and curriculum design. First, given that private speech engages neural mechanisms associated with thought regulation, educators should recognize it as a valuable cognitive resource, rather than viewing it as a sign of limited proficiency. Instructional environments should actively support learners’ engagement in private speech, particularly during complex or cognitively demanding L2 tasks. Second, the observed proficiency-dependent differences in the neural characteristics of private speech suggest a developmental trajectory that can inform differentiated instructional strategies. For lower-proficiency learners, whose private speech primarily supports language processing networks, classroom activities might encourage the use of private speech to assist with lexical retrieval and phonological rehearsal while reducing the overall cognitive load of tasks to avoid overburdening limited working memory resources. Conversely, for higher-proficiency learners, whose private speech recruits both language-related and thought-regulation networks, curriculum design can incorporate more cognitively demanding and open-ended tasks. These tasks may support the internalization of private speech into inner speech. By strategically fostering private speech as a transitional mechanism, instructional practices can be better aligned with learners’ cognitive and linguistic development, thereby promoting the functional development of private speech.

## 4. Conclusions

Using fNIRS, the present study is the first to investigate the neural mechanisms underlying private speech in L2 learners. While prior neurolinguistic research has employed fMRI to explore the neural correlates of inner and outer speech, and L2 research has primarily examined private speech from a behavioral perspective, our study bridges these domains to provide a cognitive neuroscience account of language internalization in the context of L2 learning.

Theoretically, beyond demonstrating the behavioral utility of private speech in L2 oral production, this study provides novel neurobiological evidence that both corroborates and refines core tenets of Vygotsky’s sociocultural theory. Our findings revealed that private speech enhanced functional connectivity between the language network and the thought-regulation network—similar to inner speech—thus supporting the Vygotskian view of private speech as a tool for cognitive regulation. At the same time, private speech retained connectivity features characteristic of outer speech, reinforcing its conceptualization as a transitional form between outer and inner speech. Furthermore, as L2 proficiency increases, private speech appears to evolve from primarily supporting language processing to additionally facilitating non-linguistic cognitive control. This finding enriches our understanding of the developmental trajectory of private speech within the sociocultural framework, suggesting that L2 learners at different proficiency levels engage private speech in qualitatively distinct ways.

Pedagogically, these findings advocate for instructional practices that acknowledge and support the use of private speech in L2 classrooms. Teachers should not only permit but actively encourage private speech, tailoring task design to learners’ proficiency levels. For instance, lower-proficiency learners may benefit from using private speech to support vocabulary access and phonological encoding, while higher-proficiency learners may use it to regulate higher-order, cognitively demanding tasks.

The present study has several limitations. First, the sample size was relatively small, which may have led to an underestimation of effect. Future research should aim to include a larger sample to enhance statistical power. In typical fNIRS studies, more than 20 participants per group are recommended to obtain stable and generalizable results, and, empirically, a sample size of 30 to 40 per group is often considered sufficient to detect subtle or less robust neural effects. Second, we did not conduct an analysis of behavioral data nor explore their relationship with the neural measures obtained in this study. Future research could incorporate integrated analyses of neural and behavioral data, as well as potentially include additional modalities (e.g., eye tracking, motor behavior) to better elucidate the distinct contributions of private speech and other speech types to L2 task performance. Third, the present study employed a cross-sectional design, which limits the ability to examine the longitudinal development of private speech and its associated neural mechanisms within individuals. Longitudinal designs would allow researchers to track intra-individual changes over time, providing deeper insight into the dynamic role of private speech in the process of L2 acquisition. Finally, this study focused exclusively on Chinese-speaking learners of English. Given that private speech may be shaped by cultural and linguistic factors [17], future research should pursue cross-linguistic and cross-cultural comparisons to assess whether the neural mechanisms identified in Chinese L2 learners of English generalize to learners from different language backgrounds and cultural contexts.

In sum, this study represents the first attempt to examine the neural mechanisms of private speech in L2 learners, laying the groundwork for future research: (1) We provide the first neural evidence supporting Vygotsky’s theory that private speech serves both as a scaffold for thought regulation and as a transitional stage between outer and inner speech. (2) We demonstrate that the neural function of private speech evolves with L2 proficiency, shifting from supporting language-specific processing to facilitating higher-order, non-linguistic thought regulation. (3) We offer the preliminary validation of fNIRS as a tool for investigating naturalistic private speech, which is often challenging to capture with other neuroimaging techniques, thereby highlighting its methodological value for advancing sociocultural theory research in L2 learning.

## Figures and Tables

**Table 1:** Means and standard deviations for private speech measures.

Measures	High Proficiency	Low Proficiency
U	99.750 (42.278) ^1^	106.500 (33.269)
W_PS	714.250 (371.590)	671.125 (216.766)
U/S	0.166 (0.070)	0.178 (0.055)
W/U	6.857 (1.800)	6.424 (1.341)

^1^ Means are presented outside parentheses, with standard deviations in parentheses.

**Table 2:** Means and standard deviations for oral production measures.

Measures		Inhibition	Private Speech Users		Non-Users of Private Speech	
			High	Low	High	Low
Fluency	W_OP	Free	311.313 (106.71) ^1^	300.000 (99.416)	287.813 (62.778)	283.563 (83.814)
		Inhibited	282.000 (127.436)	277.125 (100.601)	294.313 (72.621)	289.000 (112.140)
	AS	Free	22.938 (8.790)	27.813 (9.354)	21.938 (5.196)	23.375 (7.898)
		Inhibited	23.000 (9.913)	27.000 (8.892)	24.688 (7.245)	24.688 (9.700)
Complexity	CAS	Free	7.500 (2.733)	7.250 (5.209)	7.625 (4.455)	6.250 (3.088)
		Inhibited	6.813 (3.563)	5.625 (3.810)	8.125 (3.364)	7.375 (4.365)
	W/AS	Free	14.024 (2.982)	10.874 (1.258)	13.357 (2.074)	12.426 (1.930)
		Inhibited	14.021 (7.584)	10.358 (1.546)	12.225 (2.255)	12.108 (3.106)
Accuracy	EW	Free	30.188 (19.181)	29.125 (19.131)	21.313 (12.700)	30.250 (10.208)
		Inhibited	27.250 (18.237)	30.000 (23.232)	20.375 (10.059)	26.938 (11.066)

^1^ Means are presented outside parentheses, with standard deviations in parentheses.

## Data Availability

The data that support the findings of this study are available from the corresponding author upon reasonable request due to privacy.

## Abbreviations

The following abbreviations are used in this manuscript:

L2	Second language
STG	Superior temporal gyrus
IFG	Inferior frontal gyrus
MTG	Middle temporal gyrus
SMG	Supramarginal gyrus
DLPFC	Dorsolateral prefrontal cortex
fNIRS	Functional near-infrared spectroscopy
U	Number of utterances
W_PS	Number of words (during private speech)
U/S	Average number of utterances per second
W/U	Average number of words per utterance
CAF	Complexity, accuracy, and fluency
W_OP	Number of words (during oral production)
AS	Analysis of speech
W/AS	Average number of words per AS unit
CAS	Complex analysis of speech
EW	Number of erroneous words
HbO	Oxygenated hemoglobin
HbR	Deoxygenated hemoglobin
tHb	Total hemoglobin
FDR	False discovery rate
CH	Channel
OFC	Orbitofrontal cortex
AG	Angular gyrus
